# Supplementary material for: A canonical neural mechanism for behavioral variability
Source: Nat Commun. 2017 May 22;8:15415. doi: 10.1038/ncomms15415 (PMC5458148; doi:10.1038/ncomms15415)
Supplement: Supplementary Information — Supplementary Figures and Supplementary Notes, and Supplementary References [file ncomms15415-s1.pdf]

Supplementary Note 1.

A Network **Mechanism** Generating Correlated Irregular Activity

Asynchronous strong temporal irregularity and spatial heterogeneity in spiking activity of single neurons emerge naturally from the dynamics of recurrently connected neural circuits, in which strong excitation is balanced by strong inhibition (Fig.1b. Supplementary Figure 1a-b; *1-3*). In this regime, neurons exhibit irregular firing as well as large trial-to-trial variability even in the absence of *any* source of noise external to the network. The essence of this temporal irregularity arises from the fact that each neuron receives a large number of 'strong' (*1-3*) excitatory and inhibitory presynaptic inputs that cancel each other to the leading order, but the set of inputs is different for each neuron. The latter heterogeneity in the network connectivity results in temporal irregularity of the neuronal activity.

The temporal time scale on which fluctuations in the inputs or outputs of the neurons decorrelate depends on the synaptic dynamics. The theory predicts that for fast synapses this decorrelation happens rapidly (Supplementary Figure 7; (*1-2)*). By contrast, if synaptic interactions are slow (compared to the neuronal integration time) the neuronal activity exhibits slow chaotic rate fluctuations (*4-5)* (Supplementary Figure 7). In both cases, the distribution of the temporal average firing rates of the neurons is well approximated by a log-normal distribution *(6)* (Supplementary Figure 1c). The theory- derived in a completely random networks- also predicts that the averaged pair-wise spatial correlations of the neuronal activity are very weak, on the

order of $\frac{1}{N}$ where N is the number of neurons *(3,7-8)* (Supplementary Figure 1d-e). Moreover, in this regime single neuron activity is highly sensitive to temporal fluctuations, as well as to heterogeneities in the recurrent or feedforward connectivity or in small variations in external inputs. These features endow the network with remarkable computational capabilities; e.g., the emergence of selective neuronal responses to stimuli *(9)*. These conclusions, combined with experimental results(*3,10-15*), support the idea that the *balanced regime* is a fundamental mode of operation of local cortical networks.

Our paper addresses a fundamental question; namely to what extent the intrinsic temporal variability in recurrent networks can be exploited to drive motor variability. In a more general mechanistic perspective, we investigate how single neuron variability can result in uncertainty in behavior (e.g. in making a decision *(16)*). The fact that in the balanced regime neurons are only weakly correlated should apparently prevent the transfer of internally generated variability at the neuronal level to any downstream system which sums the activity of many fluctuating neurons. In this work we demonstrate that this is not the case. Specifically, we show that spatial correlations can emerge if the circuit generating and transferring the variability to the effectors has a topographic organization. As a result, the circuit can eventually produce robust exploratory behavior. In fact, we show that this mechanism does not require *any fine-tuning of parameters*. In particular, it is robust to the number of neurons N, the number of connections K, as well as to the connectivity in the topographic pathway to the effectors (and the number of neurons projecting to an effector M).

1.1 Balance of excitation and inhibition in strongly recurrent networks

To formulate in a minimal mathematical description the concept of the balanced regime, we consider an unstructured network model of one excitatory (E) and one inhibitory (I) binary neurons receiving strong feedforward inputs from an external population of excitatory neurons *(1-2)*. Following van Vreeswijk and Sompolinsky*(1-2)*, the total synaptic inputs into the excitatory and inhibitory neurons can be written:

$$H_E(t) = K(J_{EE}\nu_E(t) + J_{EI}\nu_I(t) + J_{E0}\nu_0) + \delta H_E(t)$$

$$H_I(t) = K(J_{IE}\nu_E(t) + J_{II}\nu_I(t) + J_{I0}\nu_0) + \delta H_I(t)$$

where we denote by $\delta H_\alpha(t)$, $\alpha \in \{E, I\}$ subleading (in large K) contributions to these inputs, which in particular include the temporal fluctuations. Note that the sign of the synaptic strength of the inhibition is negative ($J_{\alpha I} < 0$). The variance of these fluctuations is:

$$\sigma_E^2 = K(J_{EE}^2\nu_E(t) + J_{EI}^2\nu_I(t) + J_{E0}^2\nu_0)$$

$$\sigma_I^2 = K(J_{IE}^2\nu_E(t) + J_{II}^2\nu_I(t) + J_{I0}^2\nu_0)$$

Here, $\nu_E(t)$, $\nu_I(t)$ and ν_0 are the population-averaged firing rates of the E and I neurons and of the external population; $J_{\alpha\beta}$, $\alpha, \beta \in \{E, I\}$ and $J_{\alpha 0}$ are the strengths of the recurrent and feedforward synapses (considered for simplicity to be homogeneous for each synapse type). We also assume that the average number of synapses per neuron, K, is the same for the two populations and for both the recurrent and feedforward synapses and that it is large (the number of synapses is typically 100 or more). Scaling the strengths of the recurrent and feedforward synapses as:

$$J_{\alpha\beta} = \frac{\bar{J}_{\alpha\beta}}{\sqrt{K}} \quad (1)$$

the temporal fluctuations remain finite when K becomes large. Requiring that the mean inputs also remain finite implies:

$$\sqrt{K}(\bar{J}_{EE}\nu_E(t) + \bar{J}_{EI}\nu_I(t) + \bar{J}_{E0}\nu_0) = \mathcal{O}(1) \qquad (2)$$

$$\sqrt{K}(\bar{J}_{IE}\nu_E(t) + \bar{J}_{II}\nu_I(t) + \bar{J}_{I0}\nu_0) = \mathcal{O}(1)$$

and therefore, for large number of synapses, K, $\bar{J}_{EE}\nu_E(t) + \bar{J}_{EI}\nu_I(t) + \bar{J}_{E0}\nu_0 = 0$ and $\bar{J}_{IE}\nu_E(t) + \bar{J}_{II}\nu_I(t) + \bar{J}_{I0}\nu_0 = 0$ .These two linear equations express the fact that, in the two populations, the net inputs into the neurons are comparable to their firing threshold in spite of the fact that taken separately, excitation and the inhibition are much larger than the threshold. These two linear equations uniquely determine the population average firing rates ν_E and ν_I of the excitatory and the inhibitory populations. The requirement that ν_E, ν_I are positive, yields a set of inequalities which determines the domain of the parameters (synaptic strengths and external inputs) in which the balanced state exists *(1-2)*. Thus, recurrent networks of E and I neurons can operate in the balanced regime for a wide range of parameters without *any fine-tuning* of these parameters. Importantly, because of the scaling of Eq.(1), the amplitude of the temporal fluctuations in the inputs are comparable to the threshold even if K is large. This is why in the balanced regime neurons fire very irregularly. Similar arguments hold for rate-based units *(4)*, as well as for more realistic neuronal models, such as integrate-and-fire or conductance-based neurons *(9)*.

It is important to note that although the average rates of the E and I populations are a linear function of their inputs (see Eq.(2)), the system is highly non-linear and the output of each

neuron, as well as the dynamics of sub-populations of neurons (as we will show below), are a non-linear function of their inputs.

1.2 Irregular feedforward inputs shared by all neurons in the motor network does not drive correlated variability

Transfer of temporal variability from the premotor network downstream to the effectors requires that the motor network operates in the balanced regime. Is it also sufficient? To address this question we investigated the case, where all neurons in the motor network share common inputs from the same group of premotor cells (Supplementary Figure 2a). One might expect that this common feedforward component would tend to synchronize the activity of all the neurons in the motor network. This is not the case. As depicted in Supplementary Figure 2c, the strongly irregular activity of the neurons in the motor network only exhibits very small spatial correlations ($CV_{eff}^2 = 0.03$). In fact, the strength of the spatial correlations vanishes in inverse proportion to the average number of recurrent connections in the motor network, K ($CV_{eff}^2 \sim \frac{1}{K}$; Supplementary Figure 2b-d). This feature is a hallmark of unstructured and strongly connected recurrent networks, where the recurrent inhibition in the motor network *suppresses* the correlations due to shared inputs*(3,7)*. This can be understood heuristically as follows. As long as the matrix $J_{\alpha\beta}$, which characterizes the strength of the interactions in the motor network, is regular (see Eq.(2)) it fully determines the population average firing rates, ν_E and ν_I, as a function of the population average activity of the premotor network (ν_0). Therefore, since the latter is constant, ν_E and ν_I must be constant up to small fluctuations. Hence, the motor network cannot exhibit synchronous activity and neuronal variability cannot be transferred to the effectors.

1.3 Motor variability emerges if the premotor-to-motor projections are topographically organized

If the premotor-to-motor projections are topographically organized, the order $\sqrt{K}$ time averaged feedforward inputs to the neurons in the motor network are the same for all functional groups in the motor network. However, their temporal fluctuations, which are of $\mathcal{O}(1)$, vary from one group to the other. We found that in this case irregular activity with robust spatial correlations emerges in the motor network.

To intuitively understand why this is the case, consider a circuit with two functional groups (Supplementary Figure 5). Denoting by $\nu_{E,l}(t)$, the time-dependent population average activity of the E population in group l= 1,2, the balanced equations (Eq.(2)) yield two sets of equations for the three populations (one I and two E populations):

$$\sqrt{K}\left(\bar{J}_{EE}\frac{\nu_{E,1}(t)+\nu_{E,2}(t)}{2}+\bar{J}_{EI}\nu_I(t)+\bar{J}_{E0}\nu_0\right)=\mathcal{O}(1) \qquad (3)$$

$$\sqrt{K}\left(\bar{J}_{IE}\frac{\nu_{E,1}(t)+\nu_{E,2}(t)}{2}+\bar{J}_{II}\nu_I(t)+\bar{J}_{I0}\nu_0\right)=\mathcal{O}(1)$$

Here we assumed, as in the spiking model we simulated, that the recurrent connectivity is statistically homogeneous over the entire motor network and that the groups are defined solely by the topographic organization of the premotor FF inputs.

The balanced equations imply that $\frac{\nu_{E,1}(t)+\nu_{E,2}(t)}{2}$ and $\nu_I(t)$, the average of the instantaneous population activities of the two groups and the inhibitory population, are constant in time. Therefore, $\nu_{E,1}(t)$ and $\nu_{E,2}(t)$ can vary in time, without breaking the balance of excitation and inhibition, provided that they vary in a push-pull manner and are thus negatively correlated.

Numerical simulations indicate that this indeed occurs (analytical proofs will be presented elsewhere). The network recurrent dynamics self-organize such that $\nu_{E,1}(t)$ and $\nu_{E,2}(t)$ both exhibit significant temporal variations which are driven by the $O(1)$ shared fluctuations present in the feedforward inputs. Importantly, the temporal fluctuations in the population activities, $\nu_{E,1}(t)$and $\nu_{E,2}(t)$, are large and negatively correlated, in spite of the fact that the shared FF inputs are only weakly correlated between the two groups. This result in an average CV_{eff}^2 which is large (Supplementary Figure 5bbottom $CV_{eff}^2 \sim 0.25$) and which is finite (of order unity) even for large N, K and M (Supplementary Figure d-f).

Self-organization of the dynamics also occurs when the number of groups, D, is larger than 2 (Fig.2, Supplementary Figure 3-4). This gives rise to a spatiotemporal pattern of activity in which the firing of the neurons is positively correlated within each of the groups, while the instantaneous firing rate averaged over all the excitatory neurons in the network is constant in time (the E and I population-averaged instantaneous firing rates are constant in time, up to small fluctuations due to finite size effects in K and N).

1.4 Timescales of the fluctuations in the motor network

Our behavioral data shows that vocal babbling temporally decorrelates on a timescale of several tens to a few hundred milliseconds, depending on the species (Fig.7). Moreover, our electrophysiological recordings in singing finches demonstrate that the activity of neurons in RA fluctuates slowly and decorrelates on similar timescales (Fig.5g-i, Fig.6b-c). These timescales are significantly slower than the typical single neuron integration time, raising the question of the origin of such slow timescales.

One possibility is that the recurrent synapses in the motor network are sufficiently strong and their dynamics are sufficiently slow to generate slow chaotic rate fluctuations *(4)* (Supplementary Figure 7b, black curve). However, these fluctuations would be very weakly spatially synchronized (Supplementary Figure 7c, black curve). Thus, spatiotemporal correlations on such slow timescales between neurons projecting to the same effector would be weak and therefore unable to induce highly variable motor behavior (Supplementary Figure 7d, black curve).

Another possibility is that the fluctuations in the shared component of the *feedforward* premotor-to-motor input are slow, resulting in spatially *and* temporally correlated fluctuations at the level of population in the motor network (Supplementary Figure 7c- red and blue curves and Fig.6). This occurs naturally if the premotor-to-motor pathway involves a sufficiently large fraction of slow synapses, e.g. synapses mediated by NMDA receptors, that would low-pass filter the fluctuations generated in the premotor network. This mechanism is depicted in Fig.6 and Fig.7A.

The recurrent dynamics in the premotor network can also contribute to the slowness of the fluctuations in the shared FF inputs to the motor network and therefore to the slow decorrelation

of the motor behavior. If the recurrent synapses in the premotor network (the synapses in the E-I-E loop or of the mutual-inhibition) are strong and slow, the dynamics of the premotor neurons will be chaotic (Supplementary Figure 7a- red and blue curves) and their activity will decorrelate on a timescale on the order of the typical (and slow) synaptic time constant*(4)*.

Supplementary Note 2.

Cross-correlations in Electrophysiological Recordings in Zebra Finches

2.1 Spatial structure of the correlations in RA

In line with our model, the data reported in Figure 5, exhibited positive as well as negative cross-correlations (CCs). However, the majority of the CCs were positive. One should note that our experimental technique for recording single unit activity in singing finches is probably biased toward pairs of neurons located at rather short distances compared to RA diameter. Unfortunately, we cannot accurately report the distance between recorded neurons for two reasons. First, the electrodes (tungsten microwires) are slightly flexible and the distance between their tips (around 100 μm before being moved in the brain) can vary considerably as they are advanced down into RA. Second, post-hoc histological examination was carried out at the end of the experiment, after the electrodes had been advanced several times (with 4 electrode each time), making it impossible to associate a given electrode tract with a given recording. As RA is topographically organized (17-18), it is very likely that our bias to record from nearby neurons resulted in having many pairs recorded from the same functional group. Our theory can be further validated by showing that pairs of neurons which are far apart, and thus belong to

different functional groups, are in general more negatively correlated than more proximal neurons, which are presumably in the same group. As explained above, our single unit data cannot be used for such a validation. Instead, we recorded LFPs in RA of singing zebra finches with fixed implanted electrode micro-arrays (similar to Utah arrays) that had 7 recording sites arranged (with ground and reference electrodes) in a 3x3 lattice with 100 µm minimal distance between electrode tips. As electrodes cannot be moved after implantation, recording single-unit activity from a very dense nucleus such as RA is unfortunately impossible using this technique. However, we were able to carry out noise correlation analysis on LFP signals as we did for single or multi-unit activity. As depicted in Supplementary Figure 6b, LFPs recorded from more distant electrodes were negatively correlated, whereas when the electrodes were closer, positive and negative correlations were observed. Therefore, neurons that were recorded by two electrodes 100 µm apart or less are likely to be more positively correlated, as depicted in Fig.5, than neurons that are far apart, in line with model predictions.

2.2 Correlations measured in RA are not an artifact of a misalignment or variation in duration of the syllables and song motifs

Our electrophysiological data show stronger noise correlations in RA than in LMAN. As the length of the song motif varies slightly fromrenditionto rendition, misalignment of song-related neuronal activity could lead to spurious correlations, especially in RA which neuronal activity is known to be locked to song (see for example Fig.3).To avoid such spurious correlations we carefully time-warped our spiking data from single and multi-unit recordings based on the timing of single syllable events in the motif produced by the birds (see Material and Methods). An example of how such time-warping changed the precision of song-related firing is illustrated in Supplementary Fig. 6c. As is apparent, Supplementary Fig. 6c clearly shows that spikes are more aligned to the song motif following the time warping procedure. To assess the contribution of time jitter from behavioral misalignment to the noise correlations in RA, we re-calculated the noise correlations without any time-warping. Surprisingly, there was very little change in the level of correlation with and without time-warping.

We also estimated how much misalignment and bad time-warping could contribute to spurious correlations by shifting together the spikes of the two simultaneously recorded neurons presented in Fig.5h on each rendition by a time jitter in the range 0-500ms (Supplementary Figure 6d). The correlations did increase with time jitter, as expected, but this onlyoccurred at very large jitters above 150ms. Interestingly, the amount of correlations between the two neurons hardly changed when the jitter was up to 50 ms, far more than the estimated elasticity of the zebra finch song (around 5ms at most for a given syllable, see (19)). Therefore, for typical jitters, misalignment of the motifs did not contribute to the noise correlations measured in RA.

Supplementary Note 3.

Alternative Mechanisms for Correlated Irregular Activity

Three key hypotheses underlie our work: (1) The variability of the babbling is produced by a circuit in the CNS comprising large networks of strongly coupled neurons firing a highly irregular manner (2) These fluctuations in the activity are transferred to the effectors which sum up inputs from a rather large number of neurons, on the order of 100 or more. (3) The mechanism underlying babbling should be robust, in particular with respect to variations in the connectivity parameters.

It should be noted that if we relax Hypotheses (2); i.e, if we assume that the projections to the effectors only involve a small number of RA neurons (very sparse projections to the effectors) topographic projections from LMAN to RA are nolonger necessary for the circuit to generate fluctuations in the inputs to the effectors. However, the amplitude of the latter depends crucially on the connectivity which means relaxing Hypotheses (3).

In fact, the last two hypotheses imply that fluctuations in the activity of the neurons should become correlated at some stage in the circuit before their transfer to the effectors and that this should be a *collective* phenomenon.

In all four species we considered in our behavioral study the ACE of the babbling signal in juveniles lacked an oscillatory component, and the gesture duration distribution was well approximated by an exponential. This means that the process of generating gestures during babbling is very broad band, and has no substantial oscillatory components. The

electrophysiological data suggest that the fluctuations in neural activity in RA were also very broad band without significant oscillatory components (see the autocorrelations and cross-correlations depicted in Figures 5-6). We thus need to look for network mechanisms that can account for correlated neuronal activity exhibiting strong fluctuations without having specific frequencies in the power spectrum. This is a non-trivial constraint. In fact, in previously investigated mechanisms for robust correlated activity, the latter stems from the emergence of oscillatory collective modes in the network dynamics (for review see (20)). This is the case in the mechanisms for spike-to-spike synchrony, as well as well as in those in which synchrony emerges from firing-rate instability (see e.g. (21)). In most of these models, the oscillation phase basically remains constant over many cycles, unlike what is observed experimentally. Solutions to this problem have been proposed. They all rely on synchronous chaos and lead to temporal irregular fluctuations in the population activity (22-25). However, the spectrum of these fluctuations- although broad - is also peaked in some frequency ranges (the gamma range in the papers cited above). This is because synchronous chaos emerges from a destabilization of synchronous patterns of activity which oscillate at a frequency in this range. To the best of our knowledge, our mechanism is the first to exhibit irregular synchronous activity which to a large extent is robust to changes in the number of neurons and in the average number per neuron of feedforward and recurrent connections.

We also argue that NMDA receptors (NMDAR) in the projections from LMAN to RA can account for the rather slow time constant over which babbling decorrelates. However it is also possible that low-pass filtering occurring in RA or downstream to it due to slow neuronal dynamics (such as adaptation currents) or slow muscle responses could also contribute. Such

low-pass filtering would lead to a slower behavioral output than would be predicted from the NMDAR time constant alone. However, in the case of zebra finches, the NMDAR kinetics measures in LMAN-RA synaptic inputs in juvenile zebra finches (26) fit satisfactorily with the timescale of babbling vocalization we report, suggesting minimal low-pass filtering downstream. Moreover, muscle dynamics has been reported to be fast in the bird syrinx (<5ms, see 27-28), and even if young birds have slower muscles it is hard to imagine that their dynamics would involve timescales as long as 50-100ms. This point however needs to beconfirmedexperimentally.

Supplementary Note 4.

Methods for Supplementary Figure 8

To calculate the transformation $C_g(\Delta) = F_g(C_\xi(\Delta))$ given in Supplementary Figure 8E we assumed a Gaussian Process $\xi(t)$, with $lim_{t\to\infty}\langle\xi(t)\rangle = 0$ $lim_{t\to\infty}\langle\xi(t)\xi(t+\Delta)\rangle = c_\xi(\Delta)$ and $c_\xi(0) = 1$. Consider the threshold-power-law function: $g_{\epsilon\gamma}(x) = g(x-\epsilon)\Theta(x-\epsilon)$ with $g(x) = x^\gamma$. The AC of the process { $g_{\epsilon\gamma}(\xi)\}$ is then: $\langle g_{\epsilon\gamma}\big(\xi(t)\big)g_{\epsilon\gamma}\big(\xi(t+\tau)\big)\rangle - \langle g_{\epsilon\gamma}\big(\xi(t)\big)\rangle^2 = c_g(\tau)$.The first and second moments are:

$$G_1 = \frac{1}{\sqrt{2\pi}}\int_\epsilon^\infty d\xi\, g(\xi-\epsilon)\exp\left(-\frac{\xi^2}{2}\right)$$

$$G_2 = \frac{1}{\sqrt{2\pi}}\int_\epsilon^\infty d\xi g^2(\xi-\epsilon)\exp\left(-\frac{\xi^2}{2}\right)$$

and the correlationfunction:

$$GG\left(c_{\xi}(\tau)\right) = \frac{1}{2\pi\sqrt{1-c_{\xi(\tau)}^{2}}} \iint_{\epsilon}^{\infty} d\vec{\xi} g(\xi_1 - \epsilon) g(\xi_2 - \epsilon) \exp\left\{-\frac{1}{2(1-c_{\xi}(\tau)^2)} \left[\xi_1^2 + \xi_2^2 - 2c_{\xi}(\tau)\xi_1\xi_2\right]\right\}$$

We then numerically calculate the AC: $C_g(\tau) = F_g\left(C_{\xi}(\tau)\right) = \frac{GG - G_1^2}{G_2 - G_1^2}$. Note that for $\epsilon = -\infty$ and $g(x) = x$ we get $C_{g\_}\epsilon(\tau) = C_{\xi}(\tau)$, which is the identity function of F_g, plotted in dashed line in Supplementary Figure 8E.

Supplementary References

1. vanVreeswijk, C. and Sompolinsky, H.. "Chaos in Neuronal Networks with Balanced Excitatory and Inhibitory Activity."*Science* 274.5293: 1724-726.(1996)
2. vanVreeswijk, C. and Sompolinsky, H. Chaotic balanced state in a model of cortical circuits. Neural computation 10, 1321-1371. (1998).
3. Renart, A., et al. "The Asynchronous State in Cortical Circuits." *Science* 327.5965: 587-90.(2010)
4. Harish, O., and Hansel, D. Asynchronous rate chaos in spiking neuronal circuits.*PLoSComputBiol*,*11*(7), e1004266.(2015).
5. Kadmon, J., and Sompolinsky, H. Transition to chaos in random neuronal networks. *Physical Review X*, *5*(4), 041030. (2015).
6. Roxin, A., Brunel, N., Hansel, D., Mongillo, G. and van Vreeswijk, C. On the distribution of firing rates in networks of cortical neurons. The Journal of Neuroscience 31, 16217-16226 (2011).
7. Helias, M., Tetzlaff, T., and Diesmann, M. The correlation structure of local neuronal networks intrinsically results from recurrent dynamics. *PLoSComputBiol*, *10*(1), e1003428. (2014).
8. Hertz, J. Cross-correlations in high-conductance states of a model cortical network.*Neural Computation*,*22*(2), 427-447.(2010).
9. Hansel, D. and van Vreeswijk, C. The mechanism of orientation selectivity in primary visual cortex without a functional map. The Journal of Neuroscience 32, 4049-4064 (2012).
10. Wehr, M. and Zador, A. M. Balanced inhibition underlies tuning and sharpens spike timing in auditory cortex. Nature 426, 442-446 (2003).
11. Shu, Y., Hasenstaub, A. and McCormick, D. A. Turning on and o_ recurrent balanced cortical activity. Nature 423, 288-293 (2003).
12. Tan, A. Y., Chen, Y., Scholl, B., Seidemann, E. and Priebe, N. J. Sensory stimulation shifts visual cortex from synchronous to asynchronous states. Nature 509, 226{229 (2014).
13. Destexhe, A., Rudolph, M. and Pare, D. The high-conductance state of neocortical neurons in vivo. Nature reviews neuroscience 4, 739-751 (2003).
14. Haider, B., Duque, A., Hasenstaub, A. R. and McCormick, D. A. Neocortical network activity in vivo is generated through a dynamic balance of excitation and inhibition. The Journal of neuroscience 26, 4535-4545 (2006).
15. Ecker, A. S. et al. Decorrelated neuronal _ring in cortical microcircuits. Science 327, 584-587 (2010).
16. Wimmer, K. et al. Sensoryintegrationdynamics in a hierarchical network explainschoiceprobabilities in cortical area mt.Nature communications 6 (2015).
17. Vicario, D. S. Organization of the zebra finch song control system: functional organization of outputs from nucleus robustusarchistriatalis. *Journal of Comparative Neurology*,*309*(4), 486-494.(1991).
18. Johnson, F., Sablan, M. M., &Bottjer, S. W. Topographic organization of a forebrain pathway involved with vocal learning in zebra finches. *Journal of Comparative Neurology*, *358*(2), 260-278.(1995).
19. Glaze, C. M., & Troyer, T. W. Temporal structure in zebra finch song: implications for motor coding. *The Journal of neuroscience*, *26*(3), 991-1005.(2006).

20. Wang, X. J. Neurophysiological and computational principles of cortical rhythms in cognition. *Physiologicalreviews*, *90*(3), 1195-1268. (2010).
21. Brunel, N., & Hansel, D. How noise affects the synchronization properties of recurrent networks of inhibitory neurons. *Neural Computation*,*18*(5), 1066-1110.(2006).
22. Hansel, D., &Sompolinsky, H. Synchronization and computation in a chaotic neural network. *Physical ReviewLetters*, *68*(5), 718.(1992).
23. Hansel, D., &Sompolinsky, H. Chaos and synchrony in a model of a hypercolumn in visual cortex. *Journal of computational neuroscience*, *3*(1), 7-34.(1996).
24. Battaglia, D., Brunel, N., & Hansel, D. Temporal decorrelation of collective oscillations in neural networks with local inhibition and long-range excitation. *Physical reviewletters*, *99*(23), 238106. (2007).
25. Battaglia, D., & Hansel, D. Synchronous chaos and broad band gamma rhythm in a minimal multi-layer model of primary visual cortex. *PLoSComputBiol*, *7*(10), e1002176.(2011).
26. Mooney, R., and Konishi, M. Two distinct inputs to an avian song nucleus activate different glutamate receptor subtypes on individual neurons. *Proceedings of the National Academy of Sciences*, *88*(10), 4075-4079.(1991).
27. Elemans, C. P., Spierts, I. L., Müller, U. K., Van Leeuwen, J. L., &Goller, F. Bird song: superfast muscles control dove's trill. *Nature*, *431*(7005), 146-146.(2004).
28. Elemans, C. P., Mead, A. F., Rome, L. C., &Goller, F. Superfast vocal muscles control song production in songbirds. *PloS one*, *3*(7), e2581.(2008).

Supplementary Figures

Supplementary Figure 1

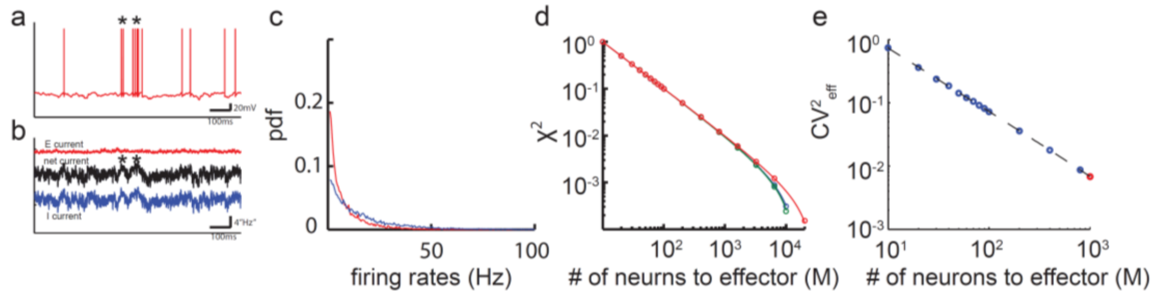

<u>Supplementary Figure 1.</u>In the balanced excitation-inhibition regime spiking is irregular, asynchronous and time-averaged firing rates are heterogeneous. Network parameters are as in Fig.1**a.**Voltage trace of one excitatory neuron in the motor network. The temporal irregularity in the action potentials is intrinsically generated by the recurrent network dynamics since there is no external noise. **b**. Total excitatory (red), inhibitory (blue) and net (E+I, black) inputs to the same neuron as in(a).The excitation and the inhibition taken separately are large relative to the threshold, but the mean and the fluctuations of the net input (black) are comparable with the threshold. Suprathreshold fluctuations in the net inputs induce irregular spikes (stars in a,b). **c.** The single neuron firing rates in the excitatory (red) and inhibitory (blue) populations are highly heterogeneous. Their distributions are long-tailed. Note that this heterogeneity stems solely from the recurrent dynamics of the network since in each population all the neurons are identical in our model. **d.** Measure of synchrony, $\chi^2(M)$, as a function of population size, M, in log-log scale (see Materials and Methods). Blue: N=10000,K=400. Green: N=10000, K=800; Red: N=20000, K=400. Blue and green lines are almost indistinguishable. In all cases $\chi^2 \sim b/M$, up to deviations for $M \approx N$. **e.**CV^2_{eff} decreases as $A + \frac{B}{M}$ with A≈0 (dashed line). Circles: simulations. Red dot corresponds to the parameters used in Fig1.

-

Supplementary Figure 2

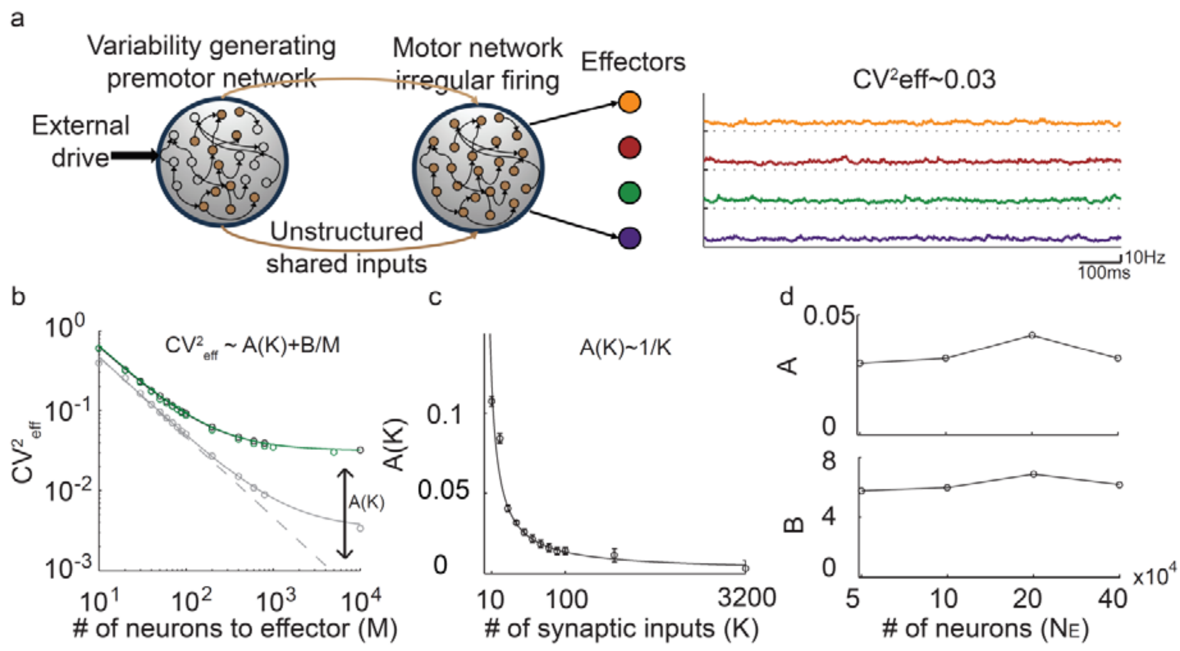

<u>Supplementary Figure 2.</u>The activity in the motor network is weakly correlated when all neurons in the motor network share the same premotor inputs. a. Left: A subset of neurons in the premotor network projects to all neurons in the motor network, thereby all neurons in the motor network share the same feedforward input. Right: The fluctuations in the inputs to the effectors are higher than in Fig.1b, but still very small ($\mathrm{CV}^2_{\mathrm{eff}} = 0.03$) and their magnitude vanishes when the number of synaptic inputs increases. **b-d.** CV^2_{eff} is well fit to $CV^2_{eff} = A(K) + \frac{B}{M}$ for large K. In (b): Green: $N_E = 40000,\ K = 400$; Black (almost coincide with green): $N_E = 10000, K = 400$; Gray: $N_E = 40000,\ K = 3200$. In (d) K=400. A and B barely depend onN_E. From (b-d) it can be concluded that$A(K) \sim 1/K$, namely the synchrony in the network is very weak.

Supplementary Figure 3

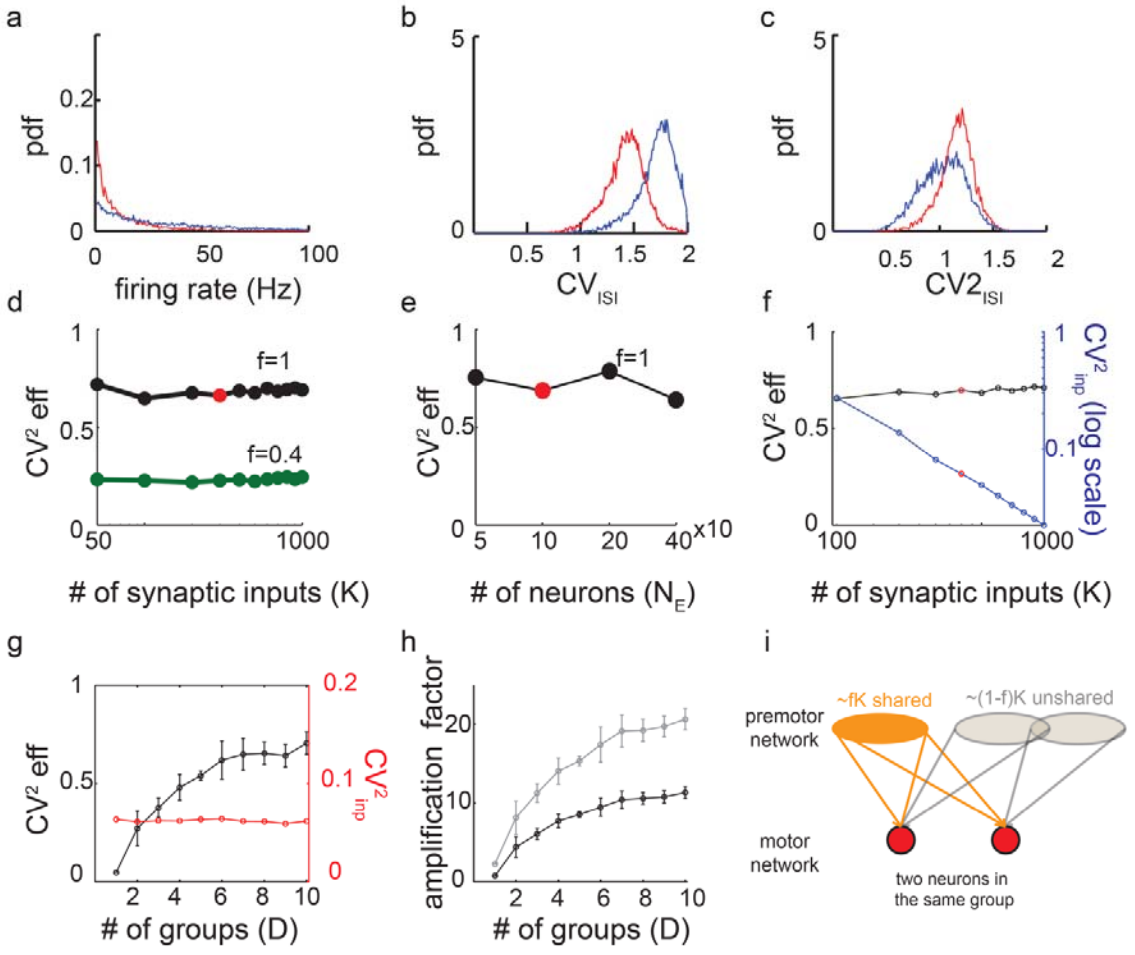

<u>Supplementary Figure 3.</u>When the circuit is topographically organized the activity in the motor network is irregular, heterogeneous and the inputs to the effectors exhibit robust temporal fluctuations. Parameters are as in Fig2.a-e. **a.** Distribution of firing rates of E and I neurons in the motor network. **b.** Distributions of CV_{ISI} for the E and I neurons. Neurons fire irregularly with high CV_{ISI}. **c.** Distributions of $CV2_{ISI} = \langle \frac{2|\Delta t_{i+1} - \Delta t_i|}{\Delta t_{i+1} + \Delta t_i} \rangle_i$ for the E and I neurons in the motor network. Here, Δt_i is the i'th ISI and the average is over all the ISIs. CV2 measures the local variability in non-stationary spike trains. Neurons are firing irregularly with CV2 ~ 1. **d-e.** The coefficient of variation of the inputs to the effectors depends only weakly on the number of connections (feedforward and recurrent)(d) andthe number of

neurons in the network(e). Red dot corresponds to the parameters used in Fig2.a-e. **f.** The CV of the inputs to the neurons (CV_{inp}^2, blue) and the inputs to the effectors (CV_{eff}^2, black) plotted vs K. CV_{inp}^2 decreases with K while CV_{eff}^2 remains essentially constant. This results in an amplification of CVs (see Fig.2g). Red dot: K=400 as in Fig2.a-e. **g.** CV_{eff}^2 (black) and CV_{inp}^2 (red) vs. number of functionalgroups.The average correlations in neuronal activity within a group increases with the number of groups. This stems from the fact that increasing the number of groups results in effectively narrowing the spatial extent of the correlations in the FF inputs with respect to the footprint of the recurrent connectivity in the motor network (see also Fg.2h-i).**h.** Amplification factor (CV_{eff}^2/CV_{inp}^2) increases with the number of functional groups. Black: $\tau_{eff} = 10ms$ as in Fig.1-2. Gray: $\tau_{eff} = 3ms.$.**i.**Cartoon of the FF projections from the premotor network to the motor network. The two neurons in the motor network are in the same functional group. By construction,the two neurons receive fK inputs from the same neurons in the premotor network (orange, shared inputs). The neurons also receive inputs drawn randomly and independently from excitatory neurons in the premotor network with probability$(1 - f)K/N$. (grey, 'unshared'). The probability that two neurons also have common inputs in this set is on the order of K^2/N^2 which is small as $N \gg K$. The overlap between the 'unshared' and 'shared' sets of inputs is not represented.

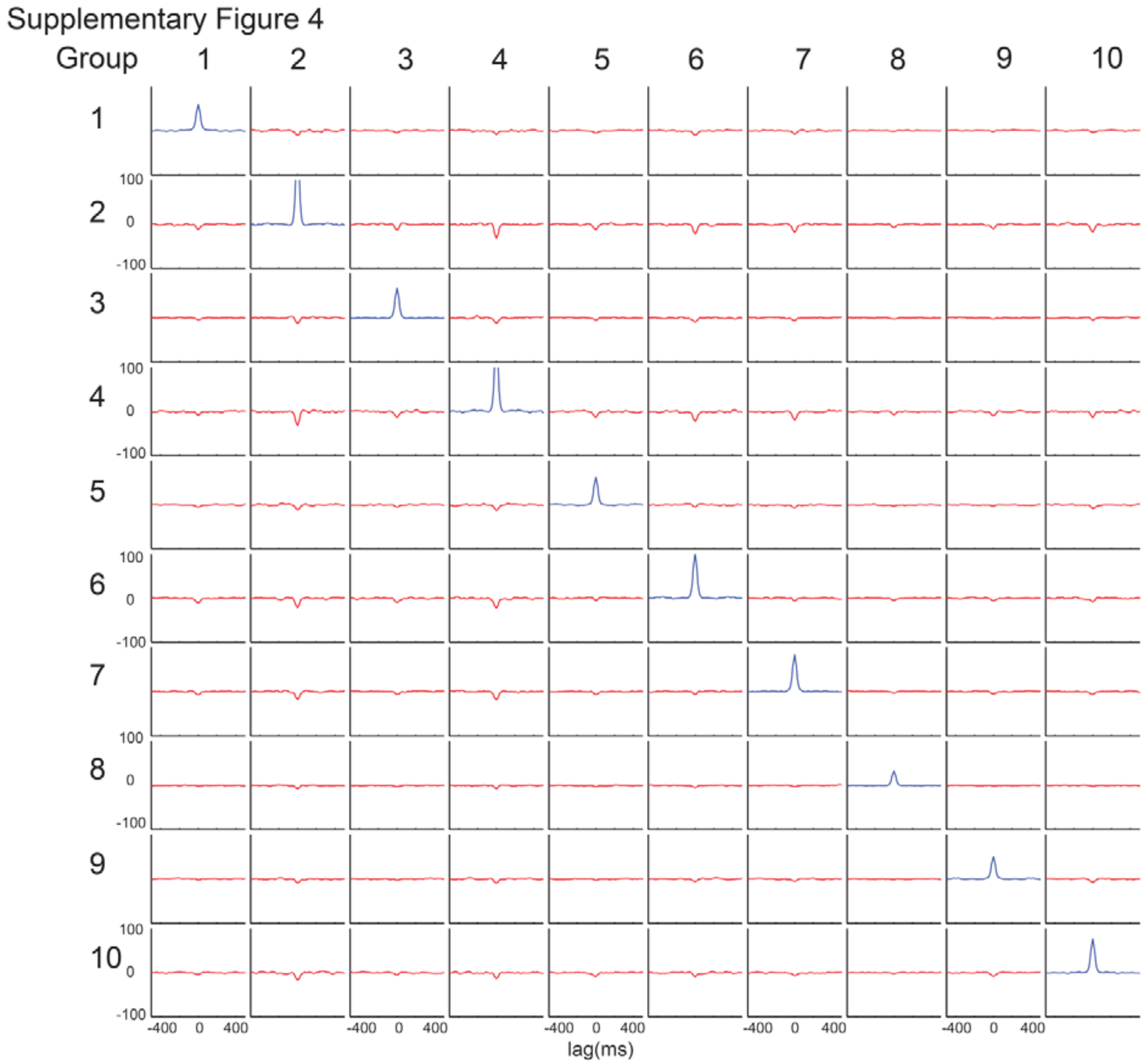

<u>**Supplementary Figure 4.**</u>**Crosscorrelations of the activity within and between functional groups in the motor network.** The population average activity of each functional group was smoothed with an exponential sliding window of 10ms. The autocorrelations (diagonal, blue; not normalized) and the crosscorrelations (off-diagonal, red; not normalized) are plotted. Note that the crosscorrelations within a group are positive and much stronger than correlations across groups, which are in general weak and negative.

Supplementary Figure 5

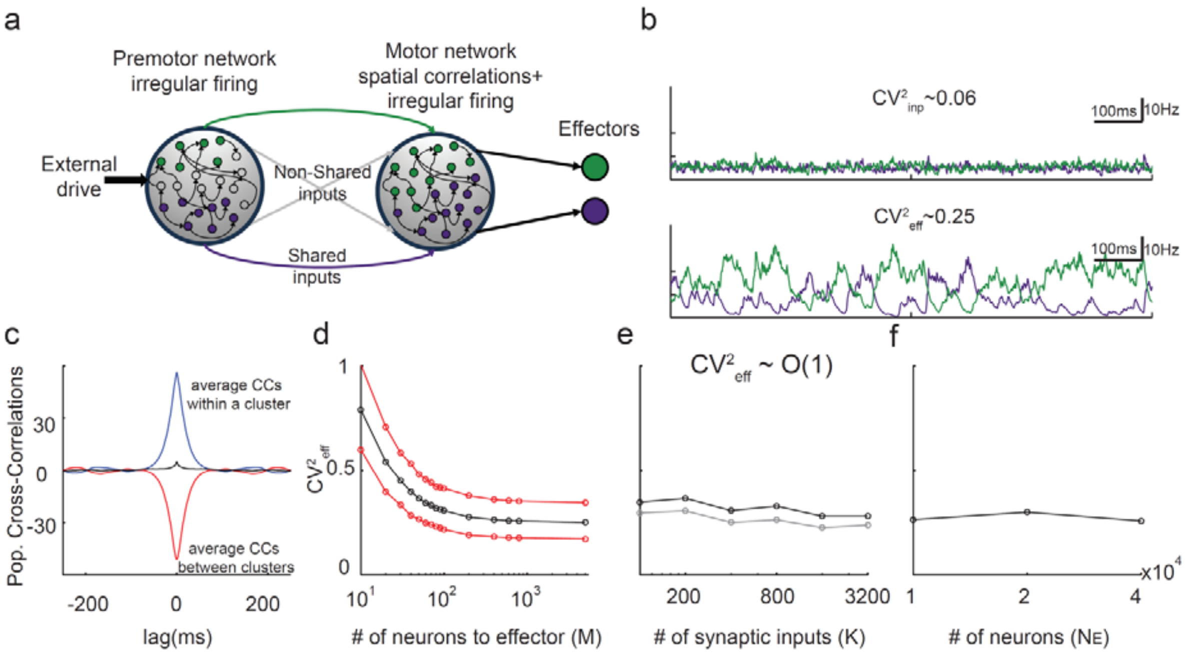

<u>Supplementary Figure 5.</u>Push-pull dynamics and spatial correlations for two functional groups.a. Architecture of the circuit.**b.** Top: Feedforward inputs to neurons in each functional group in the motor network (top). Bottom: Input to the effectors. M=1000. Note that CV_{eff}^2 is much larger than CV_{inp}^2. **c.** Population averagecross correlations for neurons projecting to the same effector are positive (blue), while average correlations between neurons projecting to different effectors are negative (red). Black: population average cross correlations over all E neurons in the motor network. **d-f.**CV_{eff}^2converges to a non-zero value, which only very weakly depends on the number of connections (e) or neurons (f) in the network. **d.** Red: CV_{eff}^2 for the two effectors; Black: average CV_{eff}^2 of the two effectors. **e.** Black: M=100 ; Gray: M=5000. **f.** M=400. Results for M=800 are similar and are not shown.

Supplementary Figure 6

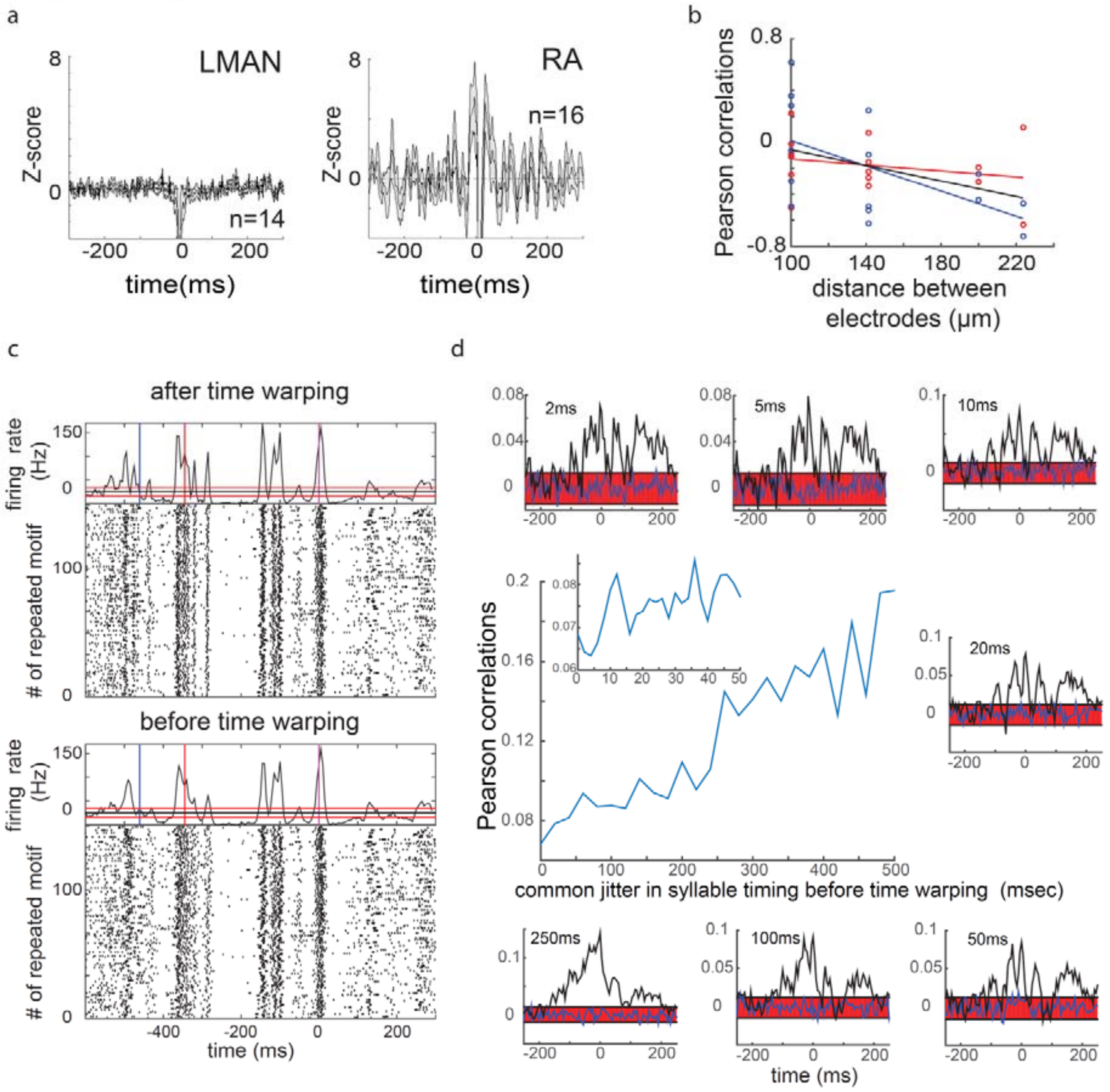

<u>Supplementary Figure 6.</u>Additional electrophysiological support for the measured correlations in LMAN and RA.a. Correlations of LMAN or RA single-units with the multi-units background activity.Left: Spike triggered average (STA) with the background multi-unit envelope in LMAN during singing. The background envelope of multi-unit activity recorded simultaneously with a single-unit on the same electrode is smoothed with a 5-ms Gaussian window. The average motif background activity is subtracted from the singing-related

background. STA with this residual signal is then computed for each single-unit recording separately, and the average z-score±s.e.m is shown. Note the ~10-ms artifact around the spike time which is due to the spike subtraction. Right: Same as in the left panel, but for RA neurons. **b**. Noise correlations between LFP recordings vs. the distance in the recorded site (see Material and Methods). Note that correlations tend to be negative between sites that are far apart. Each circle denotes a pair of recording sites, and the data were recorded in two birds (red and blue). Solid lines: Linear fit for bird 1 (red; slope -0.23; R^2=-0.056; n.s), for bird 2 (blue; slope -0.56; R^2=0.31; p=0.001) and for the two together (n=15; black; slope -0.43; R^2=0.18; p=6 10^{-14}). **c**. Raster plot of the example RA neuron also plotted in Fig.3a before (bottom) and following (top) time-warping. Note the improvement in the alignment of the spikes to the song motif. **d.** Noise correlations of the pair of RA neurons depicted in Fig.6b and 5hwithout time warping and with a common jitter (ranging from 2 to 500ms)appliedto the syllable times across renditions of the motif (see Supplementary Information). Main figure: Pearson correlations vs. the amount of jitter. Inset: Zoom-in on 0-50msjitters shows that the jitter does not dramatically change the level of correlation. Figures around the main figure: noise correlations as depicted in Fig.5h, but with an increasing time jitter (clockwise).

Supplementary Figure 7

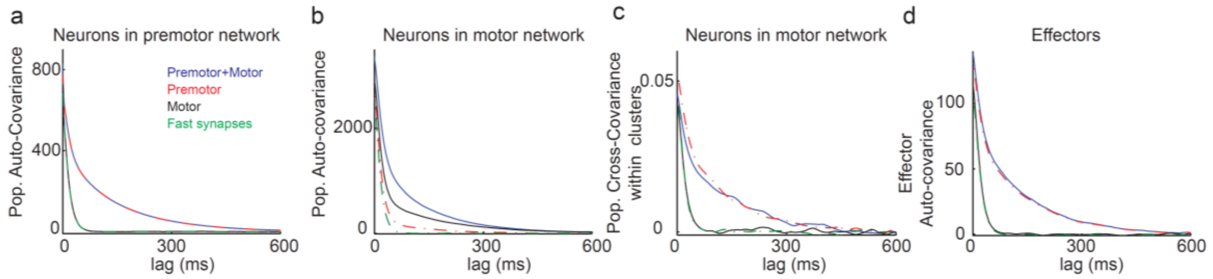

<u>Supplementary Figure 7.</u> Various mechanisms generating slow fluctuations in the motor network and in the effectors. **a.** Population average autocovariance (not normalized) of neurons in the premotor network. **b.** Population average autocovariance (not normalized) of neurons in the motor network. **c.** Population average cross-covariance (not normalized) for neurons in the motor network in the same functional group. **d.** The auto-covariance (not normalized) of the input to the effectors. In all panels the color code is as follows.Green: All synapses are fast (synaptic time constants are 3 ms) and the strength of the mutual inhibition is $\bar{J}_{II} = -4$. The decorrelation of the fluctuations is fast in the premotor and motor networks (a-b) and in the effectors (d). Red: The mutual inhibition in the premotor network has two components, one fast ($\tau_s^{II-fast}$=3ms) and one slow ($\tau_s^{II-slow}$=100ms). For both components K=400 and their strength is $\bar{J}_{II} = -2$. All other synapses are fast. The premotor network now exhibits asynchronous chaotic rate fluctuations which decorrelate on a timescale (a) on the order of the synaptic time constant of the slow inhibition[15]. Both the synchronous activity in the motor network (c) and the input to the effectors (d) decorrelate on the same timescale. Black: The mutual inhibition in the motor network has two components, one fast and one slow with the same strength, $\bar{J}_{II} = -2$.All other synapses are fast. The motor network now exhibits slow asynchronous chaotic rate fluctuations on the slow timescale of the inhibition (b) and fast synchronous fluctuations driven

by the premotor network (c). Therefore, the fluctuations in the input to the effectors (d) are fast. Blue: the mutual inhibition in both networks has two components, one fast and one slow with the same strength is$\bar{J}_{II} = -2$. In all panels $\bar{J}_{II} = -4$ in both networks, unless stated otherwise. Other parameters: $\bar{J}_{EE} = 0.3, \bar{J}_{IE} = 6, \bar{J}_{EI} = -0.8$, $\bar{I}^E = 0.2, \bar{I}^I = 0.1$ for the premotor network; $\bar{J}_{EE} = 0.5, \bar{J}_{IE} = 3, \bar{J}_{EI} = -3, \bar{J}_{II} = -4$, $\bar{I}^E = 0.2, \bar{I}^I = 0.1$; $\bar{J}_{E0} = \bar{J}_{I0} = 4$.

Supplementary Figure 8

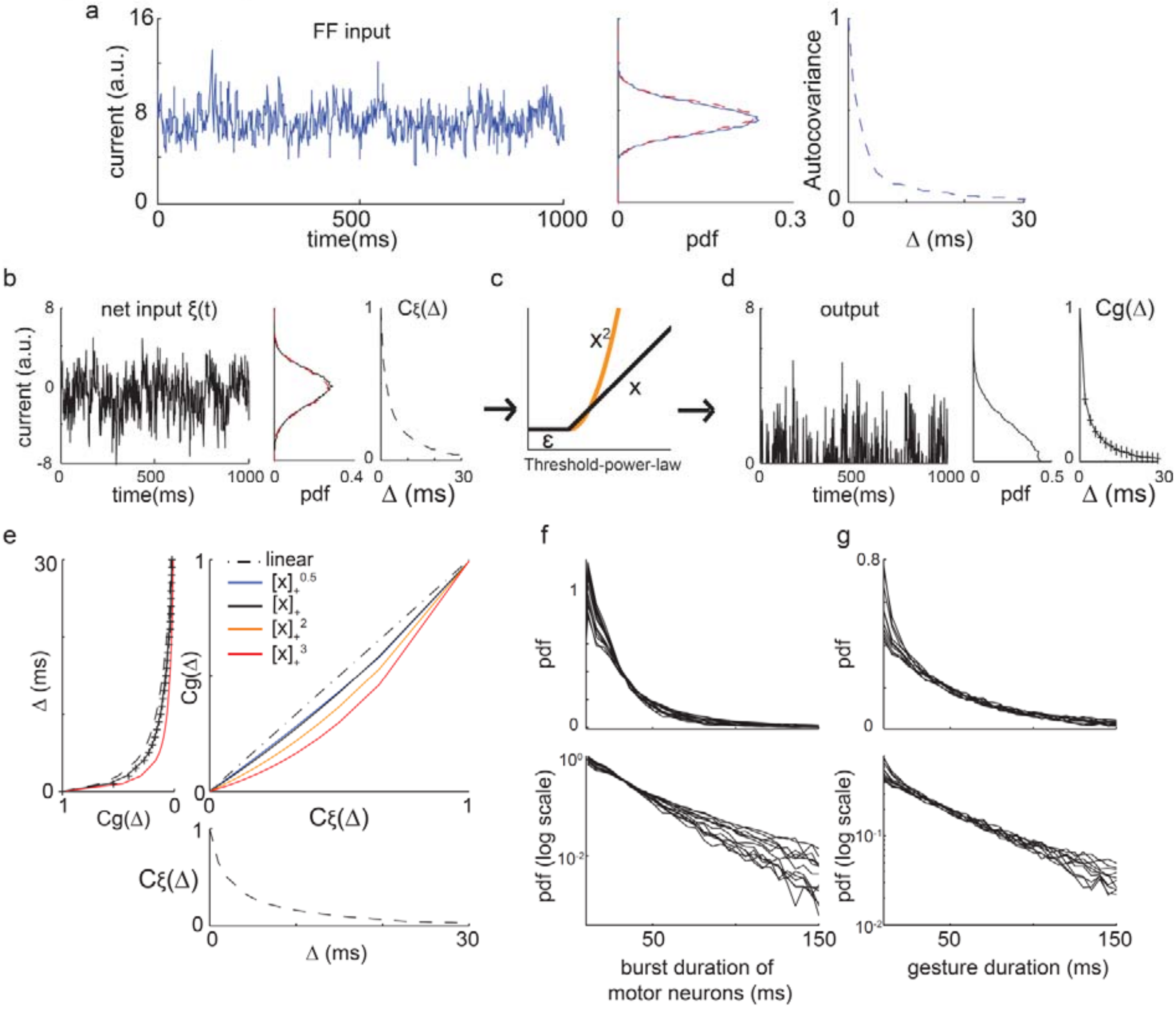

<u>**Supplementary Figure 8.**</u>**Statistics of a wideband Gaussian process after a rectified power-law transformations**. In (a), (b) and (d)**:** Left: Trace of the process. Middle: Marginal distribution of the process. Right: Autocovariance (AC) of the process depicted on the left panel. Parameters in a-b are as in Fig.7a, but with D=2. **a.** FF input to a typical neuron in the motor network. Red: The marginal distribution is well fit by a Gaussian. **b.** Net input to a typical neuron in the motor network. Red: The marginal distribution is well fit by a Gaussian (red). $C_\xi(\Delta)$: AC of the process shown in left. **c.** Rectified power-law function$g_{\epsilon\gamma}(x) = [x-\epsilon]_+^{\gamma}$. Black: $\gamma = 1$. Orange: $\gamma = 2$. **d.** The process in (b) following a rectified linear transformation:

$g_{01}(x)$. $C_g(\Delta)$: AC of the process shown in left. The pdf was estimated after excluding points smaller than 0.1. **e.** Bottom: AC of a Gaussian process $\{\xi_t\}$ with AC as in (b). Middle: Transformation between $C_\xi(\Delta)$ to $C_g(\Delta)$ for different shapes of rectified power-law functions with different exponents. Left: AC of $g_{\epsilon\gamma}(\xi)$. Dashed black: $C_\xi(\Delta)$. Black: $C_g(\Delta)$for $\epsilon = 0, \gamma = 1$. Orange: $C_g(\Delta)$for $\epsilon = 0, \gamma = 2$. Red: $C_g(\Delta)$for $\epsilon = 0, \gamma = 3$. Black crosses: AC of the process in (d). **f.** Top: Distribution of burst duration for 14 randomly chosen neurons in the motor network. Bottom: y-axis is in log scale. **g.** Top: Distribution of 'gesture' durations with a simplified thresholding of the input to the effectors ($\gamma = 1$). Bottom: y-axis is in log-scale. Parameters in f-g are as in Fig.7a and with $\tau_s^{E0} = 50ms$.

Supplementary Figure 9

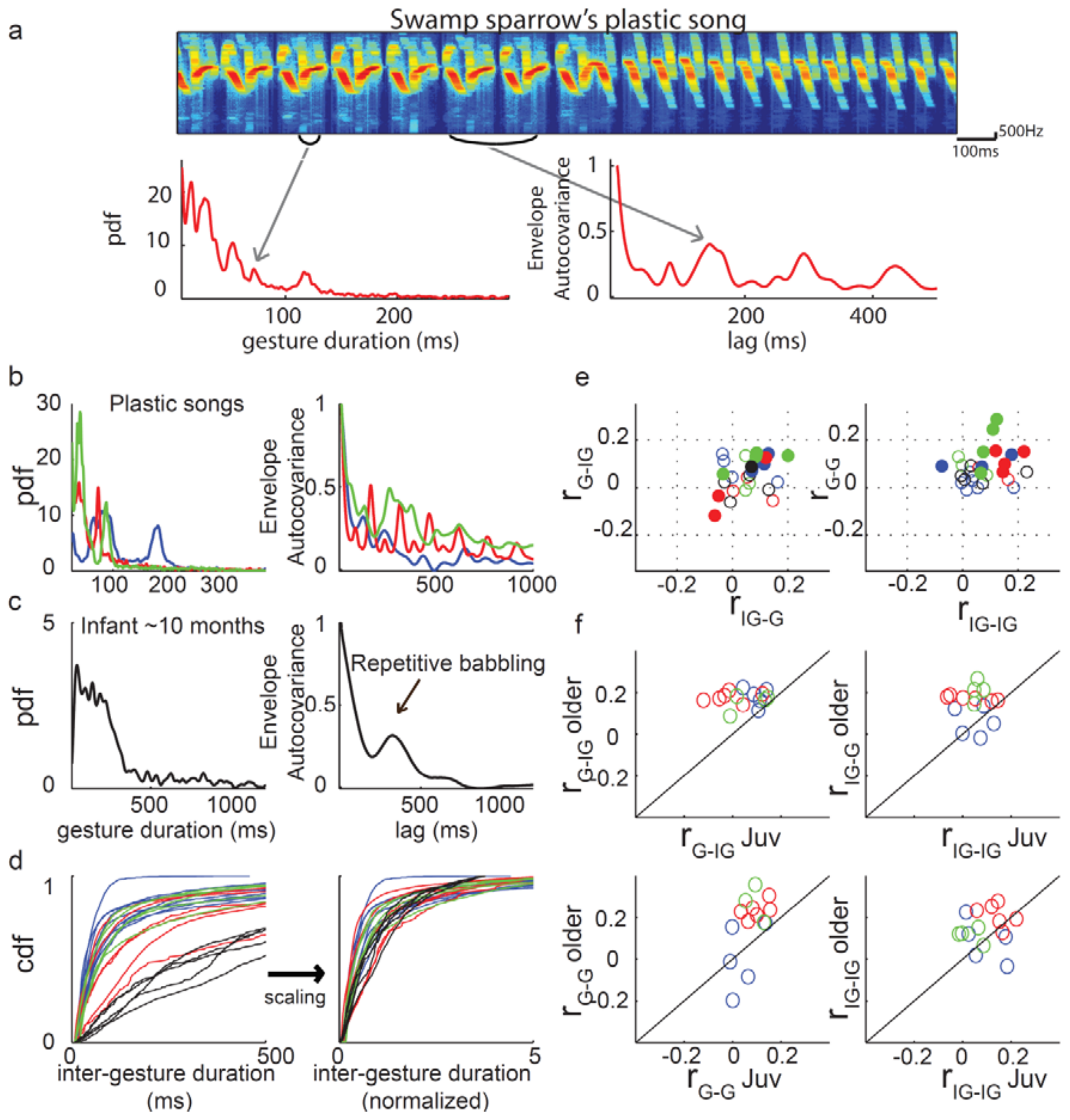

<u>Supplementary Figure 9.</u>Statistics for gesture and inter-gesture duration in young and older juveniles. Color code as in Fig.7.**a.**Temporal structure of Sw plastic song (322-364 DPH). Top: Spectrogram of vocalizations. Bottom: probability density function (pdf) of vocal gesture durations (left) and averaged auto-covariance of the envelope (ACE; right). The peaks in the distribution and the ACE express the temporal stereotypy of the song.**b.** Stereotypy of plastic

songs and diversity of songs in different species as captured by the gesture duration distributions and ACEs. Examples of gesture distribution (left) and ACE (right) of plastic songs. Note the peaks in the distributions as well as in the ACEs (Sw: 337-379 DPH; Zf: 73 DPH; Ca: 284 DPH, day post hatched). **c.** Gesture duration distribution (left) and ACE (right) of a 10 month old infant, at the beginning of a repetitive babbling period (also called "canonical babbling", with repetition of the constant-vowels, e.g. ba-ba-ba). **d.** In babbling juveniles, most of the variability between species in the CDF of the inter-gesture duration is accounted by a scaling factor of the time (as is the case for the gesture duration distributions, see Fig7.). **e.** Babbling juveniles. Left: Pearson correlations between the duration of an inter-gesture and the consecutive gesture duration (r_{G-IG}) against the correlation between the duration of a gesture and the consecutive inter-gesture duration (r_{IG-G}). Right: Same for consecutive gestures (r_{G-G}) against consecutive inter-gestures (r_{IG-IG}). Full circles: Significant non-zero correlations (for both statistics; permutation test; p<0.01). Note that in all cases correlations are close to zero with a slight tendency to be positive, probably due to global tempo changes. **f.** The Pearson correlations increase with age. Correlations for later plastic songs are larger than during babbling for the same individuals.

Supplementary Figure 10

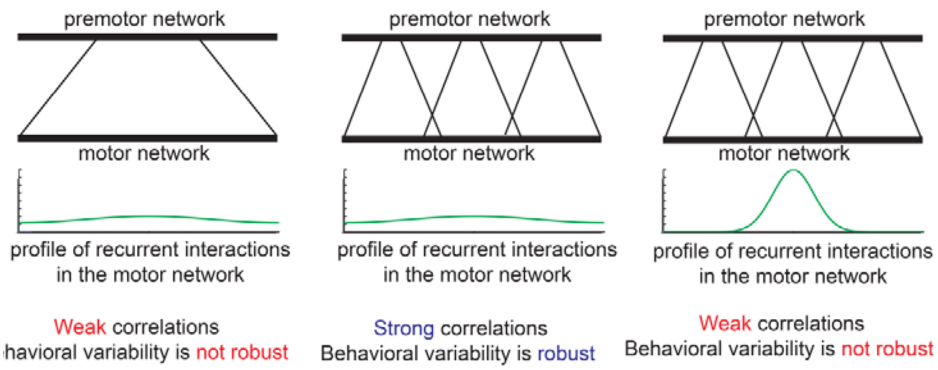

<u>Supplementary Figure 10.</u>Robust behavioral variability emerges from the interplay between topographic organization in the premotor-to-motor pathway and the recurrent dynamics in the motor network. The architecture of the premotor-to-motor pathway (top) and the footprint of the recurrent connections within the motor network (bottom) is depicted in each of the three panels. Neurons in the motor network can develop highly robust correlations when the premotor-to-motor pathway is topographically organized and the recurrent connectivity in the motor network is sufficiently wide.
